# Omani Frankincense nanoemulsion formulation efficacy and its latent effects on biological aspects of the spiny bollworm *Earias insulana* (Boisd.)

**DOI:** 10.3389/fphys.2022.1001136

**Published:** 2022-10-06

**Authors:** Mervat H. Metayi, Shimaa S. Abd El-Naby, Noha A. El-Habal, Heba H. Fahmy, Mona S. Abdou, Baber Ali, Khaled H. Abdel-Rheim, Ahmed Abdel-Megeed

**Affiliations:** ^1^ Cotton Boll Worm Research Department, Plant Protection Research Institute, Agricultural Research Center, Alexandria, Egypt; ^2^ Pesticide Formulation Research Department, Central Agriculture Pesticides Laboratory, Agricultural Research Center, Alexandria, Egypt; ^3^ Cotton Pesticides Evaluation Research Department, Plant Protection Research Institute, Agricultural Research Center, Alexandria, Egypt; ^4^ Department of Plant Sciences, Quaid-i-Azam University, Islamabad, Pakistan; ^5^ Cereals and Stored Product Insects Research Department, Plant Protection Research Institute, Agricultural Research Center, Alexandria, Egypt; ^6^ Department of Plant Protection, Faculty of Agriculture (Saba-Basha), Alexandria University, Alexandria, Egypt

**Keywords:** *Earias insulana*, Omani frankincense, nanoemulsion, *in vivo*, latent effects

## Abstract

Our research shed light on the perspective of formulation technology regarding its responsibility to provide phyto-insecticides that are worthy of research into potential novel applications. There has been an increase in interest in using nanoemulsion as a new formulation in a variety of sectors during the last several decades. *Boswellia sacra* essential oil (Fam: Burseraceae) from the resin of frankincense trees has been recently proposed as a promising ingredient in a new generation of botanical insecticides. Frankincense nanoemulsion was formulated in 5% ratios comprising frankincense oil, surfactants, and water. A frankincense nanoemulsion was prepared using a high-energy ultra-sonication process and characterized by dynamic light scattering transmission electron microscopy surface tension, viscosity, and zeta potential value. Gas chromatography/mass spectrometry (GC/MS) was used to identify the chemical profiles of frankincense essential oil. Furthermore, insecticidal effects against second instar larvae of the spiny bollworm, *Earias insulana*, as well as their latent effects on the larvae were studied. In the present study, the formulation was a good nanoemulsion. The surface tension was 53.69, the viscosity was 4.76 cPs, the zeta potential was-10 mV, and the size distribution was 41.30 nm. The polydispersity index (PDI) of the nanoemulsion was found to be 0.26, and the morphology of the frankincense nanoemulsion was visualized in a spherical shape. The main constituents identified in frankincense oil were α-pinene (15.52%); monolinolenin (12.92%); and geranylgeranyl acetate (9.99%). The results showed significant insecticidal activity against the larval stage and considerably decreased the pupation percentage with increasing the volume of the frankincense nanoemulsion. On the other hand, the latent effects of the frankincense nanoemulsion on *E. insulana* resulted in a higher prolongation of larval and pupal durations as well as a significant reduction in the weight of larvae and pupae of *E. insulana.* Additionally, frankincense nanoemulsion dramatically influenced the adult emergence percentage. It also caused a significantly lower hatchability percentage compared to the untreated control. The concentrations used and the types of mating combination have a significant effect on the fecundity of *E. insulana*. This novel frankincense nanoemulsion formulation could be used in strategies to control the spiny bollworm on cotton plants.

## 1 Introduction

Cotton (*Gossypium barbadense*) (Malvaceae) is the most significant industrial fiber crop in the world (Johnson et al., 2014), and it is used in a variety of sectors such as textiles, cooking oil, soap, and seedcake in various countries around the world. Cotton is one of the most significant commercial crops in Egypt and serves as a strategic crop by contributing to GDP (Ahmed and Delin, 2019). Approximately 166 different species of insect pests feed on cotton over the growing season, from the seedling stage to harvest, producing varying degrees and types of damage. Bollworms are the most damaging insect pests of cotton crops, and among these spiny bollworms, *Earias insulana* (Boisd.) (Nolidae: Lepidoptera) is one of the most harmful pests that attack by infesting squares, flowers, and bolls of cotton, okra, corn, and many commercial crops (Khan et al., 2007).

The spiny bollworm’s larvae preferentially consume the soft, emerging tissues of cotton plants, specifically, the terminal buds, which causes “top boring” and eventually causes the attacked flower buds and bolls to shed, resulting in significant losses in oil and lint quantity and quality (Ahmad, 1980; Atwal, 1994; Khan et al., 2007). Production is reduced by between 2.5%–6% if the incidence of spiny bollworm infection rises at a rate of 1% (Mansour et al., 1990; Al-Juboori and Ibrahim, 2000). The spiny bollworm is a mid-late season insect that mostly attacks cotton plants (El Hamaky et al., 1990). *E. insulana* is active almost all year on its various host plants and is effective almost all year on its diverse host plants (Arif and Attique, 1990; Al Shater et al., 2020). Both cotton quality and quantity are reduced as a result of these bollworm infestations. Throughout its existence, a single larva can destroy different buds and bolls. Pesticide usage for food production peaked in 2019 at about 4.19 million tons worldwide (FAOSTAT, 2021). Cotton accounts for around 50% of pesticide use in Africa (ICAC, 2019).

Growing insect resistance against insecticides has become one of the most critical problems for crop security due to the adverse effects of massively increased pesticide usage. It has already been demonstrated to be resistant to a variety of commonly used pesticides, including carbamates, pyrethroids, organophosphates, chlorantraniliprole, indoxacarb, abamectin, bistrifluron, and benzoate (Gong et al., 2021; Li et al., 2021; Shi et al., 2021). Therefore, it is critical for integrated pest management to create new-target insecticides and efficient resistance management strategies. Due to the rapid rate at which these pests are developing resistance to traditional pesticides and the high cost of treatment in cotton-producing nations, researchers have been working for the past 3 decades and are continuing to develop alternative control techniques for these pests. Due to the concern about lowering health risks and environmental pollution, natural plant extracts play a significant and effective role as pesticide alternatives (Sharma et al., 2006).

Many essential oils exhibit a variety of actions on a variety of insects, making these botanical chemicals a great resource for the creation of insecticides. Some plant active compounds exhibit stomach and contact toxicity, whereas most of them have slow-acting actions such as larval growth inhibition and interruption of insect development (Pavela, 2016; Ikbal and Pavela, 2019; Isman, 2020). The term “frankincense” refers to the oleo-gum resin made by a number of *Boswellia* species, including *Boswellia serrata*, *Boswellia sacra*, and *Boswellia frereana*, which are shrubs that grow in north-eastern Africa (Niebler and Buettner, 2016). It is still unknown whether the Egyptian frankincense came from *Boswellia papyrifera* or *B. sacra* (or both). However, there is a certain indication that *B. sacra* was used (Archier and Vieillescazes, 2000; Hamm et al., 2005), which was sourced from *B. sacra* in Somaliland, punt land (Somalia), and Oman, so the species is likely one of those traded to ancient Egypt by the land of punt, given the likely location of punt in the Eritrea-Somalia corridor.

Frankincense resin has a long history of being associated with a variety of health-enhancing properties. The resins of *Boswellia carteri* and *B. serrata* have anti-inflammatory properties and are used in traditional medicine around the globe Chevrier et al., 2005; Banno et al., 2006. Additionally, *B. serrata* extract exhibits antifungal and antibacterial activities (Weckesser et al., 2007; Salman et al., 2021; Ayub et al., 2022). In addition, extracts of gum resins from *Boswellia* spp. may have anti-cancer effects in human leukemia cell lines and animals (Winking et al., 2000; Hostanska and Saller, 2002). These findings suggest that frankincense resin contains active elements that have a favorable impact on important biological processes.

Many other studies have found that frankincense essential oil has significant insecticidal activity against pests. Subin (2021) examined the insecticidal activity of *B. carterii* oil against the third instar larvae of *Spodoptera litura* and reported that *B. carterii* oil exhibited contact toxicity. Also, Pavela et al. (2021) found that frankincense (*Boswellia* spp.) essential oils showed significant insecticidal activity against *Culex quinquefasciatus* larvae and *Musca domestica* adults, while no relevant toxicity was detected for *S. littoralis*. In contrast to this, Yang et al. (2020) found that the *B. carterii* oil did not appear to have any acute contact toxicity after 24 h of application and had moderate attraction–inhibitory activity against the adults of *Sitophilus zeamais*, which can be used in the development of new formulations of botanical pesticides. In a study conducted by Moustafa et al. (2015) using eucalyptus essential oil, the nanoemulsion was tested on larvae of *Pectinophora gossypiella* and *E. insulana* and showed greater activity than bulk oil. Also, castor oil nanoemulsion demonstrated the highest efficacy when compared to its bulk or standard emulsion, according to Abdel-Raheem (2019), who studied the effect of castor oil essential oils (bulk and nano phase) on *S. littoralis* larvae. Abd El-Naby et al. (2022) demonstrated that the prepared castor oil nanoemulsion exhibits insecticidal activity followed by castor bulk oil under laboratory conditions against the fourth instar larvae of the cotton leaf worm *S. littoralis*.

To minimize pesticide concerns, research into novel strategies, such as the application of a new self-nano emulsifying system to agricultural systems, has been necessary in recent years. The possibility of employing nanopesticides for crop protection and other prospective uses of nanoemulsions has garnered a lot of interest in recent years (Kah and Hofmann, 2014). Because of its increased water solubility of essential oils and decreased oil droplet size, the high surface area of the emulsion system allows for quick penetration of activities (Tadros et al., 2004), resulting in improved delivery to the target organisms or plants. The aim of our work is to formulate a new formulation for Omani frankincense essential oil by a high-energy method, to make it more economical in use and more realistic for its application at the industrial and commercial level, and to test its effectiveness and study the latent effect against second instar larvae of the spiny bollworm, *E. insulana*, with different volumes of the formulation. To our knowledge, no literature exists on the insecticidal properties of frankincense nanoemulsion against the second instar larvae of spiny bollworm, *E. insulana*, which stimulated our interest in investigating this further.

## 2 Materials and methods

### 2.1 Materials

Frankincense oil (*B. sacra*) was obtained from Al Barakah Factory for Herbal Oils, L.L.C, Al Maabilah Industrial Area, Seeb, Muscat, Sultanate of Oman. Scientific distributors in Cairo, Egypt, provided Tween 80. Vitamins mixture (Grand Vit with Iron Syrup), produced by Sigma Pharmaceutical industries for Sina Pharm.

### 2.2. Insect rearing

A laboratory strain of *E. insulana* (Boisd.) (Lepidoptera: Nolidae) was obtained from a laboratory colony at the Bollworms Research Department, Plant Protection Research Institute, Agricultural Research Center, Giza, Egypt. It had been reared on a semi-artificial diet for several generations away from any contamination by insecticides as described previously by Amer (2015). The experiment was performed under controlled conditions in an incubator at 26 ± 1 °C, 65 ± 5% RH, and a photoperiod 12:12 (L: D).

### 2.3 Diet preparation

To prepare the tested diets, the boiled water was added to 250 g of kidney beans and 125 g of wheat grated. It was put over heat for 70 minutes. Thereafter it was left for 20 min to cool and clarify the water from them. They were blended with 100 ml of milk in an electric blender and put in the refrigerator for 24 h. After that, other components (49 g dry active yeast, 3 g ascorbic acid, 1.75 g sorbic acid, 1.75 g methyl para hydroxy benzoate, 8 ml mixture of vitamins, 2.5 ml formaldehyde 34–38%) were added to them and blended thoroughly. Subsequently, the prepared diet was kept in the refrigerator for 24 h before using them.

### 2.4 Phytochemical profile of essential oil

The oils were stored in airtight glassware at 4°C. Chemical analyses were performed on a Gas chromatography-mass spectrometry (GC-MS) analysis. The chemical composition was performed using a Trace GC Ultra-ISQ mass spectrometer (Thermo Scientific, Austin, TX, United States) with a direct capillary column TG–5MS (30 m ^*^ 0.25 mm ^*^ 0.25 μm film thickness). The column oven temperature was initially held at 70°C and then increased by 5°C/min to 280°C with a hold of 5 min, then increased to 300 with 5 C/min. The injector and MS transfer line temperatures were kept at 250°C. Helium was used as a carrier gas at a constant flow rate of 1 ml/min. The solvent delay was 2 min and diluted samples of 1 µL were injected automatically using Auto sampler AS1310 coupled with GC in the split mode. EI mass spectra were collected at 70 eV ionization voltages over the range of m/z 40–600 in full scan mode. The ion source was set at 200°C. The components were identified by comparison of their retention times and mass spectra with those of the WILEY 09 and NIST 11 mass spectral databases.

### 2.5 Preparation of nanoemulsion (ultra sonication method)

Oil-in-water (O/W) nanoemulsion of frankincense oil was prepared in the laboratory of the pesticide formulation research department, central agriculture pesticides laboratory, agricultural research center, al-Sabahia, Alexandria, Egypt; the preparation method was used as described in a previous study by Abd El-Naby et al. (2020). Initially, a coarse emulsion of 5% oil, Tween 80, and water was made with a magnetic stirrer at 250 rpm for 10 min, which was then subjected to ultrasonic emulsification using a 20 kHz Sonicator (BANDELIN Sonopuls, Germany). Then, the formulated nanoemulsion was characterized and then stored at 4°C for further bioassays.

### 2.6 Characterization of prepared nanoemulsion

Droplet size, Zeta potential, and polydispersity index (PDI) of nanoemulsion formulations were assessed by Zetasizer Nano ZS (Malvern Instruments, Malvern-UK, 4,700 model, Germany) using a dynamic light scattering (DLS) droplet analyzer at 25 °C. The nanoemulsion sample was diluted to the required concentration using deionized water. The count rate for all diluted samples was 367.9 kcps. The morphology of the nanoformulation was visualized using transmission electron microscopy (TEM). A drop of the formulation was negatively colored with ethanol and placed on a copper grid. An electron microscope (JEOL JEM-1400Plus, Japan) with a tungsten source and an operating voltage of 80 kV was used to capture TEM micrographs.

### 2.7 Physicochemical properties of frankincense nanoformulation

#### 2.7.1 Viscosity measurement

At room temperature, the viscosity of the prepared castor nanoemulsion was measured with a “Brookfield DV II + PRO” digital viscometer (Brookfield, United States) and UL rotational adaptor (ULA), and each reading was taken after the sample was equilibrated. The viscosity (CP) of all prepared formulations was determined by reading the viscometer directly (ASTM, 2015).

#### 2.7.2 Surface tension

The surface tension of the nanoformulation of castor nanoemulsion was measured with a du Nouy Ring, a platinum/iridium ring, using Sigma Force Tensiometer 700 United States.

Before testing, the instrument should be recalibrated, and the sample being measured should be clean, homogeneous, and free of bubbles, with a stable surface. The tensiometer is used to measure surface tension (dyne/cm) (ASTM, 2014).

#### 2.7.3 pH measurement

The pH value of the undiluted nanoformulation is determined using a pH meter and an electrode system; it was measured by using a Mettler Toledo™ SevenEasy pH meter, the electrode was immersed into the sample and left for 5min without stirring during the measurement at a room temperature to allow the pH value to stabilize. The instrument must be calibrated before the measurement. The electrode was thoroughly washed between samples using a stream of distilled water to remove all traces of the previous sample.

### 2.8 Evaluation of the insecticidal activity of prepared nanoemulsion on larval mortality and different biological aspects

#### 2.8.1 Efficacy on larvae

Five grams of prepared diet were put on a Petri dish (7.50 × 2.00 cm). Different volumes of frankincense nanoemulsion of 300, 600, 900, 1200, and 1800 μL were added to the surface of the diet, while the control (treated with water only), and then left until dry. The 15-s instar larvae were transferred to a treated diet, and the Petri dishes were covered with tissue paper below the glass cover to prevent larvae from escaping, and then left to feed. Each treatment and control was replicated four times. They were incubated under controlled conditions in an incubator at 26 ± 1°C and relative humidity of 65 ± 5%. After 24 h, the alive and dead larvae were recorded for 6 days to calculate the accumulated mortality of larval stages.

#### 2.8.2 Latent effect on larvae

To study the latent effect of frankincense nanoemulsion on *E. insulana*, the alive larvae in tested volumes (300, 600, 900, 1200, and 1800 μL) of frankincense nanoemulsion and control were transferred individually to an untreated diet in glass tubes (2 × 7.5 cm) and incubated under the previous condition. The tubes were inspected daily until pupation to record larval and pupal duration, larval and pupal weight. The pupae were separated on Glass Jar (half kg.) until moth’s emergence.

#### 2.8.3 Fecundity and hatchability rates in the different mating combinations

The newly emerged moths was sexed and divided into four groups based on the combination: group one was treated males with normal females (T♂ x U ♀), group two was normal males with treated females (U ♂ x T ♀), group three was treated males with treated females (T♂ x T ♀), and group four as control was normal males with normal females (U ♂ x U ♀), each group replicated four times. The moths were fed on a 10% sugar solution. The number of deposited eggs and hatchability percentage was recorded.

The egg’s hatchability percentages were calculated according to EL-Shennawy et al. (2019) as the following equation:

$$\%\;Egg\;hatchability=\frac{no.\;of\;hatched\;eggs}{\;no.\;of\;deposited\;eggs}X\;100$$

The Fecundity percentage was calculated according to Crystal and Lachance, (1963) as follows:

$$\%\;Fecundity=\frac{no.\;of\;eggs/\;treated\;female}{\;no.\;of\;eggs/\;untreated\;female}X\;100$$

#### 2.9 Statistical analysis

All data were subjected to one-way ANOVA, followed by a Duncan multiple range test (Duncan, 1955) to determine significant differences in treatment mean values at 0.05 probability. Also, using the Abbott (1925) formula, the percentage of larval mortality was estimated and corrected.

## 3 Results and discussion

### 3.1 Chemical composition of the Omani frankincense essential oil

The GC-MS profiles for frankincense essential oil (*B. sacra*) indicating quantitative variations are listed in (Table 1). They mainly contained, α-pinene (15.52%); monolinolenin (12.92%); geranylgeranylacetate (9.99%); methyl linoleate, (9Z,11E) (2.88%); linoleic acid, methyl ester (2.88%); trans-verbenol (2.55%); cis-verbenol (2.55%); d-Limonene (2.54%); geraniol acetate (2.54); caryophyllene (2.34%); linoleic acid, ethyl ester (1.59%); verbenone (1.52%); retinol (1.40%); β-eudesmol (1.35%); palmitic acid, methyl ester (1.20%); thujone (1.18%); β -selinene (1.18%); cembrenol (1.14%).1-heptatriacotanol (1.14%). In light of the results, α-pinene was the most abundant compound found. Our results are in tune with the findings of Woolley et al., 2012; Niebler and Buettner, 2015; Subin, 2021; and Pavela et al., 2021. In contrast, Al-Harrasi and Al-Saidi (2008) found that E-β-ocimene 32.3% and limonene 33.5% were the predominant compounds, and in their analysis α-pinene constituted only 5.3%.

**TABLE 1:** Major composition (% area˃1) of *B. sacra* essential oil.

Chemical name	Formula	Area %	MW
α-Pinene	C10H16	15.52	136
Monolinolenin	C21H36O4	12.92	352
Geranylacetate	C12H20O2	9.99	332
Methyl linoleate, (9Z,11E)	C19H34O4	2.88	294
Linoleic acid, methyl ester	C19H34O2	2.88	294
trans-Verbenol	C10H16O	2.55	152
cis-Verbenol	C10H16O	2.55	152
d-Limonene	C10H16	2.54	136
Geraniol acetate	C12H20O2	2.54	196
Caryophyllene	C15H24	2.34	204
Linoleic acid, ethyl ester	C20H34O2	1.59	296
Verbenone	C10H14O	1.52	150
Retinol	C20H30O	1.40	286
β-Eudesmol	C15H26O	1.35	222
Palmitic acid, methyl ester	C17H34O2	1.20	270
Thujone	C10H16O	1.18	152
beta -Selinene	C15H24	1.18	204
Cembrenol	C20H34O	1.14	290
1-Heptatriacotanol	C37H76O	1.14	536

**MW**, Molecular weight.

### 3.2 Characterization of fresh frankincense nanoemulsion

#### 3.2.1 Droplet size and polydispersity index (PDI)

**FIGURE 1:**
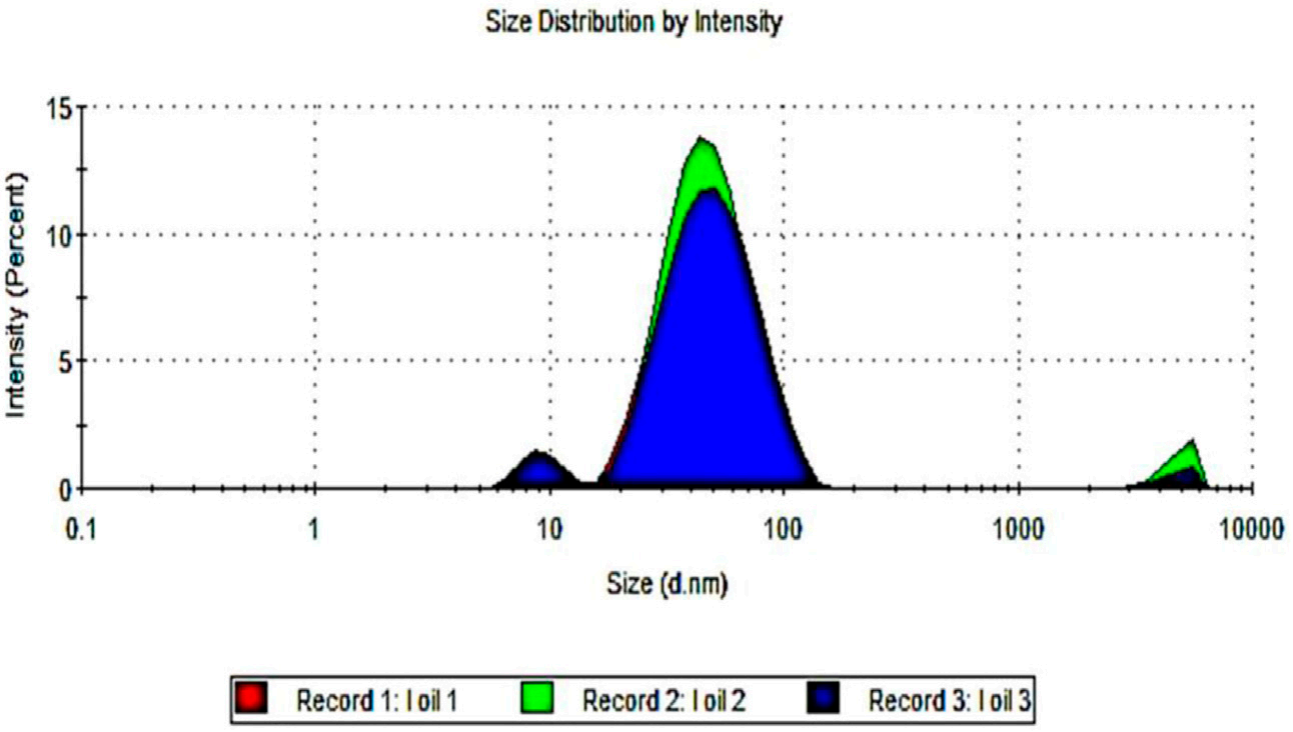
Droplet size distribution measurement by dynamic light scattering (DLS) of frankincense nanoemulsion.

The droplet size of frankincense nanoemulsion was analyzed by photon correlation spectroscopy using Zetasizer, which observes the variations in light scattering because of the Brownian motion of droplets as a function of time. frankincense nanoemulsion formulation is considered a good quality nanoemulsion Figure 1, with an average droplet size of 41.30 nm, characterizes the uniform distribution of droplets in the nanoemulsion. The PDI can range from 0 to 1, where PDI ˂0.6 stands for a nearly monodisperse system, and from our results, the PDI for frankincense nanoemulsion was 0.26As demonstrated by this finding, the nanometric size preparation for frankincense nanoemulsion was successful. Our findings support previous findings that good nanoemulsions have droplet sizes ranging from 5 to 100 nm. These results agree with a previous report by El-Mancy et al. (2021), who formulated the frankincense oil into a nanoemulsion formulation with a droplet size of 17.9 nm and a PDI of 0.2. Furthermore, consistent with findings reported by other researchers on various oils (Abdel-Rheim, (2019); Shi et al., 2012; Al-Mansoori, 2018; Abdel-Rheim, (2019); Abd El-Naby et al., 2022; Molai et al., 2022).

#### 3.2.2 Morphological analysis

**FIGURE 2:**
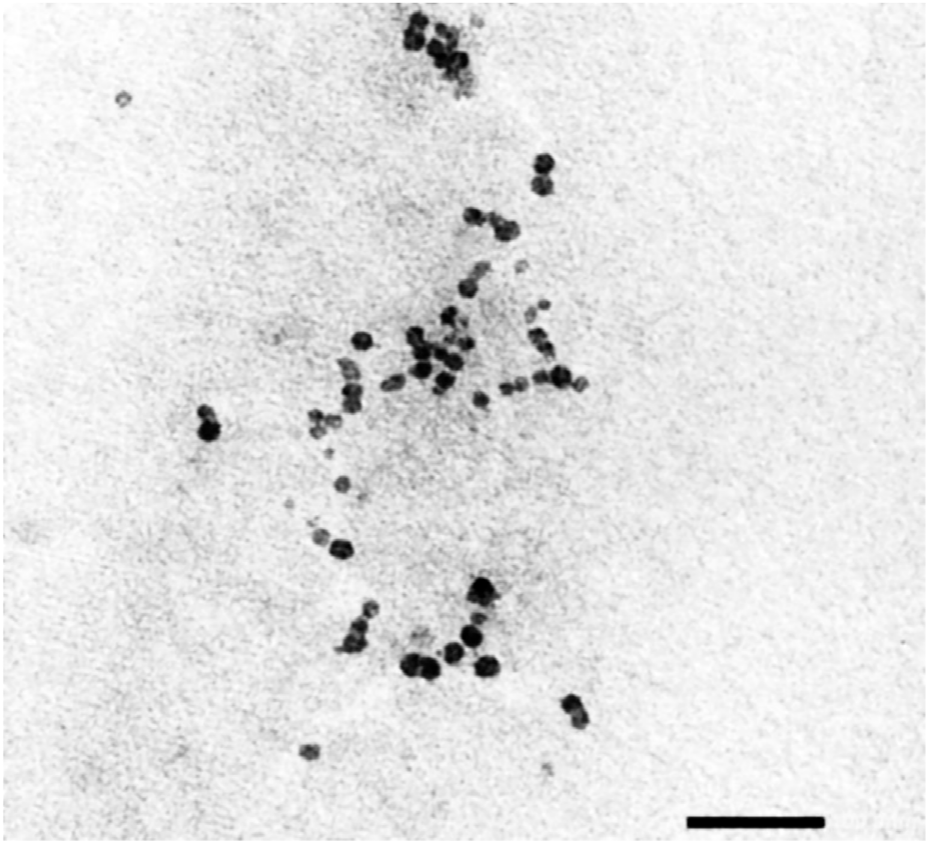
TEM image of frankincense nanoemulsion morphology illustrates spherical in shape of the prepared formulation, were taken at ×25,000 magnification (×25 Kv).

A microscopic scan of the TEM, as shown in Figure 2, reveals a spherical shape in the frankincense nanoemulsion formulation. These findings are in agreement with a prior study by El-Mancy et al. (2021), who developed a nanoemulsion formulation using frankincense oil and globules that were uniformly spherical in shape. Moreover, these findings are in agreement with findings published by other researchers on various oils (Abdel-Rheim, (2019); Abd El-Naby et al., 2020; da Silva Meira et al., 2020).

#### 3.2.3 Stability of the nanoemulsion

**FIGURE 3:**
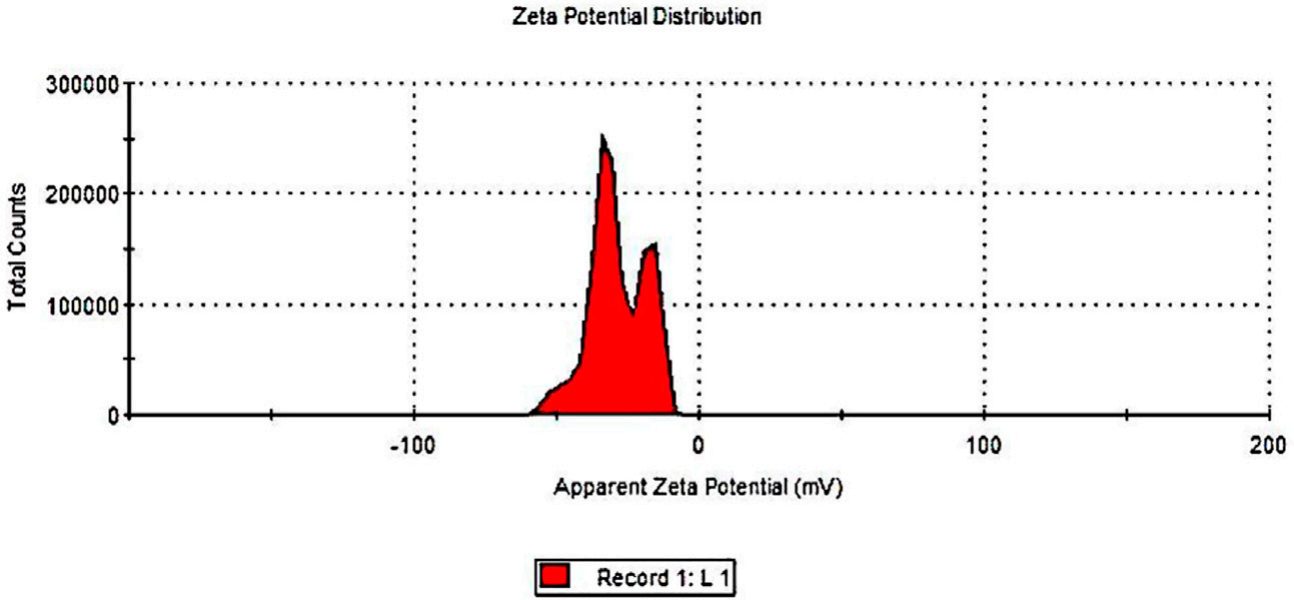
Zeta potential measurement of frankincense nanoemulsion using Malvern Zetasizer Nano ZS.

Measuring the zeta-potential value is one method for determining the physical stability of emulsion dispersion stability. Zeta potential is used to study the chemistry involved in determining whether or not an emulsion will remain stable in the environment where it will be used (Malvern Instruments Ltd., 2005). The zeta potentials of the frankincense nanoformulation are shown in Figure 3. The zeta potential of frankincense nanoformulation was in the negative zeta potential region. The nanoformulation of frankincense has a zeta potential of −29.8 mV. Colloidal stability for a variety of zeta potentials from 0 to 5 shows rapid coagulation or flocculation; from 10 to 30 shows incipient instability; from 30 to 40 shows moderate stability; and from 40 to 60 shows good stability (Kumar and Dixit, 2017). An increase in the zeta potential indicates a decrease in the attraction between droplets and an increase in the repulsive force. These results agree with a previous report by El-Mancy et al. (2021), who formulated the frankincense oil into a nanoemulsion formulation with a zeta potential value of 14.5 mV.

### 3.3 Physicochemical properties of frankincense nanoformulation

#### 3.3.1 Viscosity measurement

The determined viscosity of frankincense nanoemulsion was 4.76 cP. The viscosity of nanoemulsion formulations was generally low, which is consistent with nanoemulsion formulation properties (Ali et al., 2014; Kotta et al., 2015; Heydari et al., 2020). According to Jadhav and Nagarkar (2021), the formulation has a viscosity of about 27 mPa s, which makes it perfect for spreading and spraying on crops. Additionally, reduced viscosity improves drug absorption and speeds up drug diffusion and dissolving rates (Kollipara and Gandhi, 2014; Zhang et al., 2018).

#### 3.3.2 Surface tension

The surface tension value for frankincense nanoemulsion was 53.69 dyne/cm, which was much lower than the surface tension of water (72 dyne/cm). Decreasing in surface tension of the spray solution gave a prediction of increasing its wettability and spreading on the treated plant surface. This may lead to increased pesticidal efficiency Tadros et al. (2004). These results are completely accord with those obtained by Moustafa et al. (2015), who measured the surface tension of the prepared *Eucalyptus* nanoemulsion at 29.31 mN/m. According to Jadhav and Nagarkar (2021), the Neem and Karanj nanoformulations had a surface tension of 32.45 mNm^−1^, which was considerably less than that of water. According to Mesbah et al. (2022), the *Pelargonium graveolens* nanoemulsion surface tension value was 32.56 dyne/cm.

#### 3.3.3 pH measurement

The most important parts of chemical stability are performances in accelerated storage and the kinetics of pH profiles. The frankincense nanoemulsion showed an acidic pH value. The pH value of the prepared formulation was 6.45. Result is in agreement with Anjali et al. (2012) who reported that the pH of neem nanoemulsion formulation was 5.60, and this result was consistent with the findings of Mesbah et al. (2022). The *P. graveolens* nanoemulsion had an acidic pH value of 3.75. Furthermore, the formulation having an acidic character implies that it will have good biological activity (Issa et al., 2000; Moustafa et al., 2015).

### 3.4 Insecticidal activity of the frankincense nanoemulsion against the second instar of *E. insulana* larvae

The results as shown in Table 2 revealed that the accumulated mortality percentages of *E. insulana* increased significantly more than untreated larvae. The highest average percentage of larval mortality was recorded at 80.00% at 1800 μL compared with other volumes, while, the lowest mortality percentage was 13.33% at 300 μL compared with other volumes. To the best of our knowledge, no literature exists on the insecticidal properties of frankincense nanoemulsion against the spiny bollworm, *E. insulana*. Our findings are consistent with those of many other studies that have found that nanoformulations exhibit important insecticidal effects on other pests. According to Al Shater et al. (2020), silver nanoparticles (AgNPs) have shown significant toxic effects against the cotton spiny bollworm *E. insulana*, and as a consequence, AgNPs treatment is thought to be superior to plant extract alone for controlling *E. insulana*. Another study using the essential oil of *Eucalyptus globulus* was carried out by Moustafa et al. (2015). They tested the nanoemulsion against larvae of *E. insulana* and *P. gossypiella*. They discovered excellent effectiveness in suppressing cotton bollworms. In other work, Abd El-Naby et al. (2022) found that castor oil nanoemulsion was the most effective, followed by castor bulk oil under laboratory conditions against the fourth instar larvae of the cotton leaf worm *S. littoralis*. Another potential application of frankincense nanoemulsion was proposed by Al-Mansoori (2018) frankincense nanoemulsion had the strongest inhibitory effects against *Listeria monocytogenes* as an antibacterial. Marouf et al. (2021) indicated that camphor nanoemulsion is more efficient than camphor essential oil against *S. Littoralis*.

**TABLE 2:** Effect of formulated frankincense essential oil on larval mortality, pupation percentage, and adult emergence percentages of *E. insulana*.

Tested compound	Concentrations μL	Accumulated mortality of larval stages (%)	Pupation (%)	Adult emergence (%)
**Frankincense nanoemulsion**	300	13.33 ± 0.45^c^	82.29 ± 0.96^ab^	81.60 ± 1.28^a^
	600	20.00 ± 0.55^c^	81.60 ± 1.26^bc^	75.00 ± 0.96^ab^
	900	33.30 ± 0.89^b^	65.00 ± 1.71^c^	61.60 ± 3.97^b^
	1200	73.30 ± 1.14^a^	23.30 ± 2.38^d^	21.60 ± 1.03^c^
	1800	80.00 ± 1.52^a^	16.60 ± 0.58^d^	15.00 ± 0.66^c^
**Control**	0	0.00 ± 0.00^d^	96.60 ± 0.58^a^	93.30 ± 1.63^a^
**LSD 0.05**	—	1.18	2.07	2.87

Within the same column and source data followed by the same letter are not significantly different (*p* > 0.05); LSD, mean separately.

On the other hand, all volumes caused a significant reduction in pupation percentage compared with the control, as shown in Table 2. When compared to the control, frankincense nanoemulsion caused a significant decrease in pupal percentages of *E. insulana*. The highest pupal percentage was recorded at 82.29% with frankincense nanoemulsion at 300 μL as compared with 96.60% in control, while the lowest one was 16.60% with frankincense nanoemulsion at 1800 μL. Analogous results were reported by Ammar and Abd-ElAzeem (2021), who reported that the treatment with gelatin-copper nanoparticles released the lowest values of LC_50_ and caused the highest values of pupal mortality and pupation of the spiny bollworm. Attia et al. (2020) assess the effects of mesoporous silica nanoparticles, cinnamon oil encapsulated with silica nanoparticles, cinnamon oil, and silica gel on several biological parameters of *Corcyra cephalonica* sixth instar larvae. When compared to the control, pupation percentage, pupal duration, adult emergence percentage, and adult longevity were reduced in all treatments. Also, Kandil and El-shenawy (2018) determined that treatments had a significant effect on pupation percentage since both bug oil and sesame oil caused a reduction of 29% and *Thuja orientalis* and α-pinene caused a reduction of 30, and 35%, respectively, while it recorded 93% for the control group. In contrast, Gaaboub et al. (2012) found that the pupation percentage of *S. littoralis* fourth instar larvae treated with jojoba oil increased.

In addition, all volumes of the frankincense nanoemulsion as shown in Table 2 caused a significant reduction in *E. insulana* moth emergence. The average percentage of adult emergence was 93.30% in the control. This average decreased significantly to 15.00% at volume 1800 μL. At adult emergence, there was a statistically significant difference between the various concentrations of frankincense nanoemulsion and the control. These results corresponded with the findings of El-Din et al. (2020), who indicated that jojoba oil had a highly significant reduction in the percentage of adult emergence. El-Shewy (2018) found that using the highest concentration (0.1%) of jojoba nanoemulsion produced a highly significant decrease of 15.30% for adult *Agrotis ipsilon* emergence in comparison to jojoba free oil 3%, it produced a 34.32% reduction for adults emergence. Also (El Bendary and El-Healy, 2013; Borie et al., 2014), found that when *S. littoralis* neonates were treated with hydrophobic nano-silica, biological parameters such as adult longevity were reduced.

### 3.5 Biological effects of the frankincense nanoemulsion formulation against *E. insulana*

#### 3.5.1 Larval and pupal durations

Data in Table 3, illustrates the latent effect of tested volumes on the larval and pupal duration of *E. insulana*. The larval duration was recorded 14.80, 15.60, 17.60, 17.70, and 18.50 days at volumes of 300, 600, 900, 1200, and 1800 μL respectively, compared with 14.20 days in control. In the same trend, pupal durations were registered as recorded 7.25, 7.30, 7.11, 7.50, and 7.90 days at volumes of 300, 600, 900, 1200, and 1800 μL respectively, compared with 7 days in control. Frankincense nanoemulsion generally caused a significant prolongation of the time needed for larvae development, which may have been caused by its unfavorable effects as a larval repellent and anti-feeder, which had a significant effect on the metabolism and some biological parameters as well as the physical activity of the larvae. Due to these latent effects, *E. insulana* has a longer pupal development time and life cycle.

**TABLE 3:** Latent effects of frankincense nanoemulsion on immature stages of *E. insulana*.

Tested compound	Conc μL	Larval		Pupal	
		**Duration** (days) Mean ± SD	**Weight** (g) Mean ± SD	**Duration** (days) Mean ± SD	**Weight** (g) Mean ± SD
**Frankincense nanoemulsion**	300	14.80 ± 0.59^b^	0.048 ± 0.038^b^	7.25 ± 0.21^a^	0.051 ± 0.064^d^
	600	15.60 ± 0.78^b^	0.047 ± 0.039^b^	7.30 ± 0.22^a^	0.050 ± 0.063^d^
	900	17.60 ± 1.09^a^	0.047 ± 0.086^b^	7.11 ± 0.39^a^	0.047 ± 0.043^c^
	1200	17.70 ± 1.10^a^	0.017 ± 0.025^d^	7.50 ± 0.77^a^	0.047 ± 0.061^b^
	1800	18.50 ± 1.16^a^	0.016 ± 0.016^d^	7.90 ± 0.57^a^	0.044 ± 0.025^b^
**Control**	0	14.20 ± 2.43^b^	0.080 ± 0.046^a^	7.00 ± 0.28^a^	0.052 ± 0.030^a^
**LSD** 0.05	—	1.96	0.070	0.67	0.075

Data are the means ± SD, of the three replicate of immature stages. Within the same column and source data followed by the same letter are not significantly different (*p* > 0.05; LSD, mean separately.

In terms of statistics, there was a significant difference found between the various volumes of frankincense nanoemulsion and the control at larval duration, but no significant difference was observed between the various concentrations of frankincense nanoemulsion and the control at pupal duration. From the results, we found frankincense nanoemulsion showed biological effectiveness against bollworms. These results are in harmony with those obtained by, Ammar and Abd-El Azeem (2021) indicated that the whole larval duration of the bollworm treated with biosynthesized nanoparticles was shorter than that of the untreated group, 11.26 days, which was the most significant difference compared to control larvae. Also, El-Shennawy and Kandil (2017) found that spiny bollworm larval duration was significantly prolonged as a result of treatment with zinc oxide nanoparticles. Kandil and El-shenawy (2018) reported that sesame oil and α-pinene increased the larval and pupal duration of *P. gossypiella.*

#### 3.5.2 Pupal and larval weight

The statistical analysis of the results given in Table 3 revealed that there were highly significant differences between the various volumes of frankincense nanoemulsion and the control. At the highest volume 1800 μL, frankincense nanoemulsion showed a significant reduction in pupal weight 0.044 as compared to the control 0.052. Furthermore, the highest volume of 1800 μL of frankincense nanoemulsion produced a significant reduction in larval weight of 0.016 as compared to the control of 0.080. The present findings indicate that all tested volumes resulted in a significant decrease in pupal and larval weight. Our results are in tone with previous research but with different types of nanomaterials and insects by El-Shewy (2018), who reported that the highest concentration of Jojoba nanoemulsion 0.1% caused a higher reduction in both larval 0.22 g and pupal 0.21 g weights of *A. ipsilon* compared with control for larvae 0.38 g and pupa 0.35 g. Additionally, Adel et al. (2014) proved that the pupae that resulted from larvae treated with the two concentrations of 1.25 and 0.625% of geranium essential oil loaded-SLNs showed a significant *p*˂0.05 underweight pupae 4.95 ± 0.18 and 5.95 ± 0.11 mg respectively compared with control pupae 7.93 ± 0.11 mg. EL-Shennawy and Kandil (2017) emphasized that when using ZnO NPs against newly hatched larvae of *E. insulana*, caused highly decrease in larval and pupal weight by approximately 10 and 13.6 times, respectively, compared to the control. In the same context, Amin et al. (2019), revealed that a significant decrease occurred in the pupal weight of *A. ipsilon* in the peppermint and neem nanoemulsion formulations in comparison with control of 410.00 mg, while the mean pupal weight for the treatment of neem nanoemulsion was 359.17 mg and 273.57 mg for peppermint nanoemulsion.

#### 3.5.3 The latent effect of frankincense nanoemulsion on the fecundity of different combinations between male and female adults

Results on the latent effect of frankincense nanoemulsion on the rate of egg production are presented in Table 4. The results indicated that when adult males that came from treated larvae mated with untreated females, the fecundity of these females decreased at volumes of 300–1200 μL which gave 75.43 to 22.86%. The reduction in fecundity was increased when high volumes were applied to adult males. A significant reduction of 5.71% occurred when adult males were treated with1800 μL of frankincense nanoemulsion. Also, from the results, when adult males and females that came from treated larvae mated, the fecundity of these females decreased at volumes of 300 μL which gave 56.00%. The reduction in fecundity was increased when high volumes of 600–1200 μL were applied to adult males. No egg laying occurred when adult males were treated with1800 μL of frankincense nanoemulsion. On the other hand, when females that emerged from treated larvae were mated with untreated males, the fecundity of such females decreased significantly when the used volume of frankincense nanoemulsion was increased. The results indicate that at volumes more than 300 μL of frankincense nanoemulsion, the fecundity was significantly reduced from 65.71 to 0.00% when compared with the control at 100%. It is noticeable from Table 4, that when the treated females were mated with untreated males, the fecundity was much lower than when the treated males were mated with untreated females. The results from the present study highlighted the inhibitory effect of frankincense nanoemulsion on fecundity and fertility of *E. insulana* with different volumes and the types of mating combination have a significant effect. Similar findings were also obtained in some previous studies with different oils on different insects (Hategekimana and Erler, 2020) revealed that the EOs had an inhibitory effect on the fecundity was seen in the eucalyptus oil treated T♀ × U♂ combination recorded 47.55% higher than U ♀ × T ♂ recorded 37.68%, also Anise oil T♀ × U♂ combination recorded 74.24% higher than U ♀ × T ♂ recorded 49.33%. In this regard, the results of Ammar and Abd-ElAzeem (2021) also indicated that there was a highly significant decrease in the percentage of hatchability and egg numbers of *E. insulana* in the case of treatment with biosynthesized nanoparticles, where gelatin-copper nanoparticles (G-CuNPs) were compared to the untreated group. In the same context, Pavunraj et al. (2020) proved that silver nanoparticles exhibit 100% oviposition deterrent activities on *E. vittella*. Results also are in the same direction as that recorded by Adel et al. (2014) who investigated the use of geranium essential oil-loaded nanoparticles against *Phthorimaea operculenlla* and confirmed that the percentage of fecundity with respect to control became low and produced a notable decrease in offspring, as well as the percentage of hatchability compared with untreated females.

**TABLE 4:** Latent effect of frankincense nanoemulsion and combination between male and female adults on total eggs laid and hatchability percentages of *E. insulana* under laboratory conditions.

Tested compound	Conc μL	Mating possibilities		Average no. of eggs/♀ (Mean ± SD)	Hatched Eggs (Mean ± SD)	Hatchability (%)	Fecundity (%)
		♀	♂				
**Frankincense nanoemulsion**	300	T	T	98.0 ± 10.4^cd^	50 ± 3.35^de^	51.02	56.00
		T	U	115 ± 2.90^bc^	70 ± 1.65^bc^	60.87	65.71
		U	T	132 ± 16.0^b^	86 ± 1.35^b^	65.15	75.43
	600	T	T	60.0 ± 5.00^fgh^	29 ± 2.35^ef^	48.33	34.29
		T	U	85.0 ± 3.35^def^	46 ± 3.35^cd^	54.11	48.57
		U	T	89.0 ± 3.35^cde^	52 ± 3.35^cd^	58.43	50.86
	900	T	T	50.0 ± 2.35^ghi^	15 ± 2.00^ghi^	30.00	28.57
		T	U	65.0 ± 2.65^efg^	33 ± 2.00^ef^	50.77	37.14
		U	T	79.0 ± 3.65^def^	43 ± 4.35^de^	54.43	45.14
	1200	T	T	25.0 ± 1.65^ijk^	6 ± 1.15^ghi^	24.00	14.29
		T	U	35.0 ± 1.95^hij^	10 ± 4.16^gh^	28.57	20.00
		U	T	40.0 ± 1.35^ghi^	14 ± 4.16^fg^	35.00	22.86
	1800	T	T	00.00 ± 0.00^k^	0.0 ± 0.0^i^	00.00	00.00
		T	U	00.00 ± 0.00^k^	0.0 ± 0.0^i^	00.00	00.00
		U	T	10.00 ± 0.65j^k^	1 ± 0.00h^i^	10.00	5.71
**Control**	0	U	U	175 ± 8.35^a^	160 ± 9.35^a^	91.40	100
**LSD** 0.05	—			9.53	5.79	—	

Data are the means ± SD, of the three replicate of immature stages. Within the same column and source data followed by the same letter are not significantly different (*p* > 0.05; LSD, mean separately. **T**- moth resulted from treated larvae; **U**-untreated normal moth.

#### 3.5.4 The latent effect of frankincense nanoemulsion on hatchability in different combinations between male and female adults

Results on the effects of the extended (latent) effect of frankincense nanoemulsion on the fertility of the adult males and females are presented in Table 4, as percentages of egg hatchability. The results indicated that frankincense nanoemulsion caused a noticeable decrease in the percentage of deposited eggs at all tested concentrations. The reduction in egg viability was significant for all three groups (T♂ x U♀), (U♂ x T♀), and (T♂ x T♀), with the greatest reduction in egg hatch occurring at a high volume of 1800 μL for both groups (T♂ x T♀) and (U♂ x T♀) no hatching. While in the (T♂ x U ♀) group, their percentages were 10.00% in comparison with 91.40% for the control.

It can be concluded that the hatchability percentage of *E. insulana* was found to depend mainly on the volumes of frankincense nanoemulsion applied and the type of sex combination. The effect on fecundity was more pronounced in the cases of treated females paired with normal males and treated females paired with treated males than when normal females were mated to treated males. These results seem to be logical since the adult female is the responsible sex for egg production. Changes in fecundity may be caused by interactions between exogenously applied EOs or their major components and the pest’s ovipositional behavior. Some earlier research reported that the lower fertility in the cross between treated females and untreated males (T♀ x U♂) is clearly related to the direct effect of plant derivatives on diverse tissues such as trophocytes, prefollicular tissue, follicular epithelium, and oocytes themselves. Similar to this, Saxena and Mathur (1976) hypothesized that the decreased fecundity caused by plant derivatives may be caused by a disruption in regulatory functions rather than a direct impact on ovarian tissue. In terms of the cross between the untreated female and the treated male (U♀ x T♂), the lower fecundity may be the consequence of damage to the germ cells, which causes the concentration of spermatozoa to liquify. It is also possible that smaller spermatophores or fewer spermatophores result in a reduced number of eggs fertilized when compared to control. Earlier investigations into various insects yielded similar results (Bořkovec et al., 1968; Kaur et al., 1993). The findings of several earlier investigations show that plant EOs have considerable effects on ogenesis, leading to decreased oviposition and that they seem to have a juvenomimetic function, similar to juvenile and molting hormone (Williams 1956; Nakanishi et al., 1966; Shah 1994). Additionally, Campolo et al. (2018) suggested that lower offspring output might be related to a drop in daily fecundity and the low viability of eggs deposited by treated females or females mated with treated males. Also, in another explanation, Hategekimana and Erler (2020) found that the pure main components reduced fecundity and fertility much less than their respective essential oils. This could be caused by some synergistic interactions between certain components of essential oil. (Dhifi et al., 2016). According to Burt (2004), using essential oils in their whole form promotes more biological activity than doing so with only their main parts.

The reduction in egg hatchability caused by nanoformulations has been reported by several authors. Ammar and Abd-El-Azeem (2021) reported that the percentage of hatchability and egg count of *E. insulana* were decreased significantly by treatment with gelatin-copper nanoparticles. Also, Adel et al. (2014) indicated that geranium essential oil-loaded nanoparticles are more effective in reducing female fecundity and the percentage of hatchability. In addition, Hassan and Thesis, (2012) showed that the effect of treatments at different concentrations of *Mentha piperita*, *P. graveolens*, and *Ocimum basilicum* oils on fecundity and fertility when normal males were combined with treated females resulted that the average number of eggs per mated female being dramatically reduced at all three plant oil tested concentrations; it was extremely significant when compared to the control. Also, Boraei et al. (2014) confirmed that the increase of silica nanoparticles concentration showed adverse effects on the cotton leaf worm biological aspects, especially at high concentrations. The percentage of hatchability affected by using different doses of silica nanoparticles was recorded as low percent compared with control.

## 4 Conclusion

Our research shed light on the perspective of formulation technology regarding its responsibility to provide phyto-insecticides that are worthy of research into potential novel applications. In this regard, in our study, we succeeded in preparing the formulation and then studying and analyzing the physical characteristics of the created nanoemulsion formulation using TEM, it was discovered that the formulation had a spherical shape. The synthesized nanoemulsion has a mean droplet size of 41.30 nm and a low polydispersity of 0.26. This study also showed the prospective potential of frankincense essential oils for developing novel insecticidal formulations against spiny bollworm larvae, *E. insulana*. For instance, under laboratory settings, the frankincense nanoemulsion had a stronger efficiency against larvae in their second instar and had long-lasting inhibitory effects on larvae, pupae, adult longevity, and reproductive capacity of *E. insulana*. Additionally, frankincense nanoemulsion could be included in strategies to control cotton pests. Considering all the potential of frankincense nanoemulsion as a drug delivery system, so we hope with this formulation to be more effective in the next study. We will develop it to enhance the control of insects as well as enhance its various agricultural applications.

## Data Availability

The raw data supporting the conclusions of this article will be made available by the authors, without undue reservation.
